# Mambalgin-2 Induces Cell Cycle Arrest and Apoptosis in Glioma Cells via Interaction with ASIC1a

**DOI:** 10.3390/cancers12071837

**Published:** 2020-07-08

**Authors:** Maxim Bychkov, Mikhail Shulepko, Dmitry Osmakov, Yaroslav Andreev, Anastasia Sudarikova, Valeria Vasileva, Marat S. Pavlyukov, Yaroslav A. Latyshev, Alexander A. Potapov, Mikhail Kirpichnikov, Zakhar O. Shenkarev, Ekaterina Lyukmanova

**Affiliations:** 1 Shemyakin-Ovchinnikov Institute of Bioorganic Chemistry, Russian Academy of Sciences, 119997 Moscow, Russia; maksim.bychkov@gmail.com (M.B.); mikhailshulepko@gmail.com (M.S.); osmadim@gmail.com (D.O.); yaroslav.andreev@yahoo.com (Y.A.); marat.pav@mail.ru (M.S.P.); kirpichnikov@inbox.ru (M.K.); zakhar-shenkarev@yandex.ru (Z.O.S.); 2 Institute of Molecular Medicine, Sechenov First Moscow State Medical University, 119991 Moscow, Russia; 3 Institute of Cytology, Russian Academy of Science, 194064 St-Petersburg, Russia; anastasia.sudarikova@gmail.com (A.S.); vasileva.valeriia@gmail.com (V.V.); 4 Federal State Autonomous Institution, N.N. Burdenko National Medical Research Center of Neurosurgery, 125047 Moscow, Russia; yaroslav.latyshev@gmail.com (Y.A.L.); info@nsi.ru (A.A.P.); 5 Faculty of Biology, Lomonosov Moscow State University, 119234 Moscow, Russia; 6 Phystech School of Biological and Medical Physics, Moscow Institute of Physics and Technology (State University), 141701 Dolgoprudny, Moscow Region, Russia

**Keywords:** glioblastoma, astrocytes, mambalgin-2, amiloride-sensitive ion channels, ASIC, cell cycle, apoptosis, Ly6/uPAR, three-finger proteins

## Abstract

Gliomas are fast growing and highly invasive brain tumors, characterized by tumor microenvironment acidification that drives glioma cell growth and migration. Channels containing Acid-sensing Ion Channel 1a subunit (ASIC1a) mediate amiloride-sensitive cation influx in late stage glioma cells, but not in normal astrocytes. Thus, selective targeting of ASIC1a can be a perspective strategy for glioma treatment. Here, ASIC1a expression in U251 MG and A172 glioma cells, but not in normal astrocytes, was demonstrated. Recombinant analog of mambalgin-2 from black mamba *Dendroaspis polylepis* inhibited amiloride-sensitive currents at ASIC1a both in *Xenopus laevis* oocytes and in U251 MG cells, while its mutants with impaired activity towards this channel did not. Mambalgin-2 inhibited U251 MG and A172 glioma cells growth with EC_50_ in the nanomolar range without affecting the proliferation of normal astrocytes. Notably, mambalgin-2 mutants did not affect glioma cell proliferation, pointing on ASIC1a as the main molecular target of mambalgin-2 in U251 MG and A172 cells. Mambalgin-2 induced a cell cycle arrest, inhibited Cyclin D1 and cyclin-dependent kinases (CDK) phosphorylation and caused apoptosis in U251 MG and A172 cells. Moreover, mambalgin-2 inhibited the growth of low-passage primary cells from a patient with glioblastoma. Altogether, our data point to mambalgin-2 as a useful hit for the development of new drugs for glioma treatment.

## 1. Introduction

Gliomas are the most common brain tumors that originate from glial cells and are characterized by fast growth and high invasiveness. The ability of gliomas to proliferate in a dense environment of brain tissue is ensured by enhanced aerobic glycolysis and significant acidification of the tumor milieu [1]. According to microelectrode pH measurements, the pH of glioma isolated from patients varies from 5.8 to 7.1, while median pH of normal patient brain tissue is about 7.1 [2,3]. Low pH of extracellular environment may lead to subsequent intracellular acidification, which drives tumor cell proliferation, metastasis and helps to escape anti-tumor immunity [4]. Tumor cells possess several pH-sensitive ion channels for the regulation of intracellular ion balance and support proliferation and cellular motility. The most sensitive channels to pH changes are acid-sensing ion channels (ASICs). ASICs are members of the degenerin/epithelial Na^+^ channel (DEG/ENaC) family activated by extracellular acidification [5,6]. To form functional amiloride-sensitive channels, six subunits: ASIC1a, ASIC2a, ASIC1b, ASIC2b, ASIC3 and ASIC4 combine into homotrimers or heterotrimers, the most common of which are ASIC1a/2a heteromer and ASIC1a or ASIC3 homotrimers [5,6]. ASICs are expressed in the nervous system and are involved in a wide range of physiological processes, including pain perception, synaptic plasticity, and memory, and are considered as potential therapeutic targets for the treatment of pain, and neurological and psychiatric diseases [5,7].

Late-stage glioblastoma (GBM) cells differ from astrocytes or early-stage glioma cells by a presence of constant cation current [8], which is mediated by channels formed by ASIC1a, α- and γENaC subunits [9,10]. Extracellular microenvironment acidification leads to the recruitment of the ASIC1/ENaC channels into a cell membrane [10], and ASIC1a-mediated intracellular cation conductance may drive glioma cell growth and migration [11]. On the other hand, ASIC1a can form the complexes with Ca^2+^/calmodulin-dependent protein kinase II (CaMKII) and integrin-β1 [12], which are important regulators of intracellular signaling and adhesion. Thus, the amiloride-sensitive cation channels can participate in the regulation of glioblastoma cell proliferation by different mechanisms. In line with that, ASIC1a is overexpressed in gliomas compared to normal brain tissue (TCGA and GTEX studies, [13]). In addition to gliomas, ASICs are involved in the regulation of lung [14], liver [15], breast [16], and other carcinomas growth, migration and drug resistance [17,18]. Thus, the targeting of the ASIC1a-containing channels by specific inhibitors could become a perspective strategy for selective glioma and other cancer therapy [7,19,20].

There are several ASIC ligands with antiproliferative activity against glioblastoma cells. For example, antidiuretic drug amiloride and its analogues inhibit both ASIC-mediated currents and the proliferation of glioblastomas in vitro [21]. Amiloride also decreases the rate of mice metastasis in vivo [22]. However, the low specificity of amiloride limits its usage in the glioblastoma treatment. Another example of a natural inhibitor of ASICs is the spider toxin PcTx1 from *Psalmopoeus cambridgei* [23], which suppresses proliferation and migration of glioblastoma cells by inhibiting the amiloride-sensitive current [11,24]. However, clinical usage of PcTx1 is limited by its ability to potentiate human ASIC1a at physiological pH [25] and ASIC1b at elevated concentrations [26]. Thus, the search and development of new ligands targeting ASIC1a and with the ability to regulate oncogenesis of glioma cells is a still high-relevant task.

Potent and specific inhibitors of ASICs, mambalgins, were isolated from black mamba (*Dendroaspis polylepis*) and green mamba (*Dendroaspis angusticeps*) venoms [27,28]. Mambalgins belong to the Ly6/uPAR protein family and are presented in three isoforms. Mambalgin-2 differs from mambalgin-1 only by one Tyr/Phe amino acid substitution in the fourth position, while mambalgin-3 also has one Thr/Ile substitution at the position 23 compared with mambalgin-1. These 57 amino acids peptides have a typical three-finger structural fold with three protruding loops emerging from the β structural core. Mambalgins effectively and selectively inhibit currents through homo and heterochannels containing the ASIC1a subunit [27], whilst failing in inhibition of the ASIC2a, ASIC3, ASIC1a/3, and ASIC1b/3 channels [27]. Presently, mambalgins are considered perspective anesthetics [27], however their potential anti-tumor properties have not been elucidated yet.

Previously, we have developed an effective system for the recombinant production of mambalgin-2, a selective inhibitor of ASIC1a containing channels [29,30]. Here, we analyzed the expression profile of different ASIC and ENaC subunits in glioma cells and normal astrocytes, and revealed the selective ASIC1a expression in malignant cells. We demonstrated the functional expression of the amiloride-sensitive ASIC1a channels in glioma U251 MG cells, and showed that mambalgin-2 inhibits these channels and growth of U251 MG and A172 glioma cells with EC_50_ in nanomolar range, but not of normal astrocytes. The decrease of glioma cell proliferation upon mambalgin-2 treatment was accompanied by a cell cycle arrest and apoptosis induction. Moreover, the growth inhibition of primary cells from a patient with GBM upon incubation with mambalgin-2 was demonstrated. Thus, we revealed and characterized a new antiproliferative activity of mambalgin-2, which could be common for other ASIC1a expressing tumors.

## 2. Results

### 2.1. Astrocytes and Glioma Cells Express Different ASICs and ENaCs

**Figure 1:**
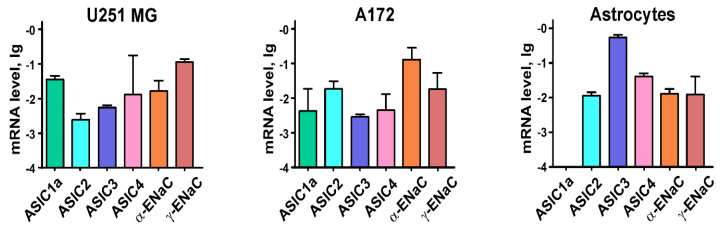
qPCR analysis of Acid-sensing Ion Channels (ASICs) and epithelial Na^+^ channels (ENaCs) mRNA expression in U251 MG and A172 cells and normal astrocytes. Gene expression was normalized to the β-*ACTIN*, *GPDH* and *RPL13a* housekeeping genes and presented as lg of relative mRNA level ± standard error of mean (SEM) (*n* = 3–5).

First, we analyzed the expression of different ASICs and ENaC subunits in glioma U251 MG and A172 cells, and normal astrocytes. qPCR analysis revealed that normal astrocytes as well as U251 MG and A172 cells express the ASIC2, ASIC3, ASIC4, α- and γ-ENaC subunits (Figure 1). The expression of ASIC3 mRNA in normal astrocytes was much higher than in U251 MG and A172 cells, whereas ASIC1a mRNA was found only in U251 MG and A172 cells (Figure 1). This means that the channels containing the ASIC1a subunit could be involved in the regulation of the various processes in malignant cells, and selective inhibitors of these channels could be used to control glioma progression.

### 2.2. Mambalgin-2 Inhibits ASIC1a in Xenopus laevis oocytes

**Figure 2:**
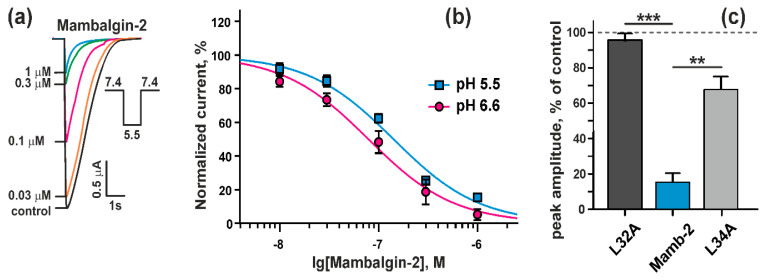
Effect of recombinant mambalgin-2 on rat ASIC1a expressed in *Xenopus laevis* oocytes: (**a**) Representative responses recorded in absence of mambalgin-2 (control) or presence of different mambalgin-2 concentrations, induced by buffer pH change from 7.4 to 5.5; (**b**) Dose–response inhibitory curves for mambalgin-2 at rat ASIC1a were fitted using Hill equation with IC_50_ 142 ± 12 nM and 79 ± 9 nM for pH 5.5 stimulus (n = 6) and pH 6.6 stimulus (n = 8), respectively. The Hill coefficient was assumed equal to 1.0. Data are presented as % of control (without mambalgin-2) ± SEM; (**c**) Comparison of the peak amplitude of the transient currents at ASIC1a at pH 5.5 in presence of 1 μM mambalgin-2 and its variants with L32A and L34A substitutions. Data are presented as normalized peak current amplitude, % of control ± SEM (n = 6). Control level (100%) is shown by dashed line. ** (*p* < 0.01) and *** (*p* < 0.0001) indicate significant difference between data groups according to One-way ANOVA followed by Dunnett’s test.

It was reported previously that the inhibitors of ASIC1a, such as amiloride and PcTx1, inhibit the proliferation of glioma cells [21,23], but demonstrate low selectivity. Mambalgin-2 from *Dendroaspis polylepis* is known as a selective inhibitor of the channels containing ASIC1a [27]. We obtained the recombinant analogue of mambamgin-2 using a previously designed *E. coli* expression system [29], and tested its activity with the two-electrode voltage clamp technique on *Xenopus laevis* oocytes expressing rat ASIC1a. Recombinant mambalgin-2 significantly inhibited the transient component of the ASIC1a currents at pH 5.5 (Figure 2a). The inhibition was reversible, because after the mambalgin-2 wash-out, the response parameters recovered completely. Mambalgin-2 at concentrations >1 µM completely inhibited ASIC1a currents at pH 5.5. The inhibitory effect was concentration dependent and fitted well with the logistic equation with the half-maximal inhibitory concentration (IC_50_) of 142 ± 12 nM (Figure 2b).

Contrarily, mambalgin-2 variants with substitutions of the residues Leu32 and Leu34 important for the toxin interaction with ASIC1a [31] demonstrated a significantly lower inhibitory activity. Mambalgin-2 at 1 µM concentration inhibited the transient component of the ASIC1a currents at pH 5.5 up to ~16% of the control, while the mutants Leu32Ala and Leu34Ala up to ~96% and ~69%, respectively (Figure 2c).

Thus, the recombinant analogue of mambalgin-2 demonstrates ASIC1a inhibitory activity close to that of the native toxin isolated from *Dendroaspis polylepis* venon [28].

### 2.3. Mambalgin-2 Inhibits ASICs Activity in U251 MG Glioma Cells

**Figure 3:**
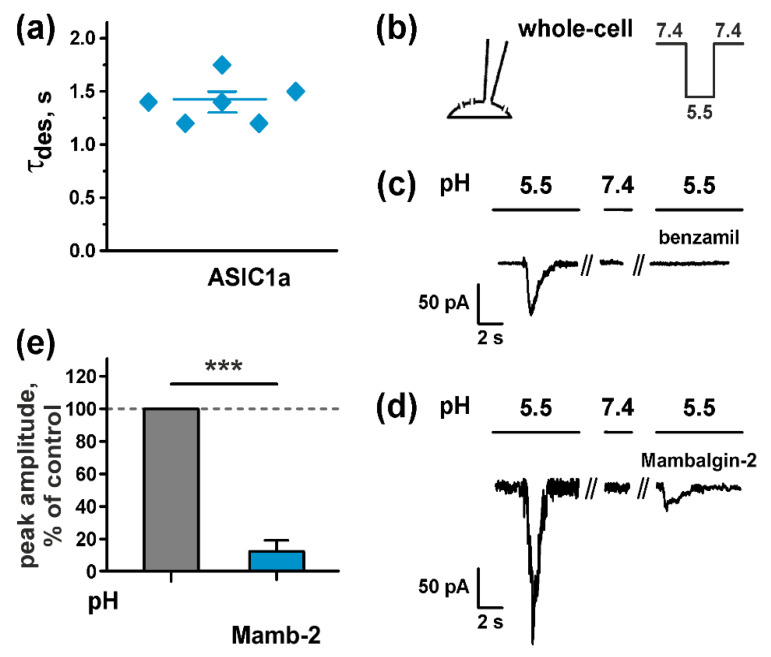
Mambalgin-2 effect on ASIC1a expressed in U251 MG glioma cells: (**a**) Desensitization time constant τdes of ASIC1a. Data are presented as mean ± SEM (*n* = 6); (**b**) Whole-cell configuration of the patch clamp technique and protocol of experiment; (**c**) Activation of the ASIC1a currents in U251 MG cells caused by rapid change of the extracellular solution pH from 7.4 to 5.5 (control, on the left), in the solution with pH 7.4 (in the middle), and upon application of 10 µM benzamil to the extracellular solution with pH 5.5 (on the right); (**d**) Activation of the ASIC1a currents in U251 MG cells caused by rapid change of the extracellular solution pH from 7.4 to 5.5 (control, on the left), in the solution with pH 7.4 (in the middle), and upon application of 1.5 µM mambalgin-2 to the extracellular solution with pH 5.5 (on the right); (**e**) Influence of 1.5 µM mambalgin-2 application to the extracellular solution with pH 5.5 on the peak amplitude of the ASIC1a currents in U251 MG cells. Data are presented as normalized peak current amplitude, % of control ± SEM (*n* = 3). Control level (100%, untreated cells) is shown by dashed line. *** (*p* < 0.001) indicates significant difference from the control according to two-sided *t*-test.

Before the study of the proposed antiproliferative activity of mambalgin-2, we tested if glioma cells express functional ASICs channels. Using the whole-cell mode of the patch clamp technique (Figure 3b) we—for the first time—characterized transient currents in U251 MG cells, induced by a rapid pH drop of the extracellular solution from 7.4 to pH 5.5. In the most cells inward currents had peak amplitude 40–312 pA (n = 11). Almost half of the cells demonstrated transient currents with the desensitization time constant (τ_des_) typical for ASIC1a (τ_des_ = 1.41 ± 0.08 s at pH 5.5, Figure 3a). Application to the U251 MG cells of amiloride analog benzamil (10 µM), the potent ASIC1a blocker with IC_50_ ~3.50 µM [32], caused an almost full inhibition of transient ASIC1a currents evoked by acidification of the extracellular solution to pH 5.5 (Figure 3c). Notably, no current through ASIC1a expressed in U251 MG cells in the extracellular solution with pH 7.4 was detected (Figure 3c). The data obtained point on the expression of the functional ASIC1a channels on the surface of U251 MG cells.

Similar to benzamil, the application of 1.5 µM mambalgin-2 (the concentration at which the toxin inhibited the ASIC1a channels expressed in *X. laevis* oocytes up to ~8%, Figure 2b) to U251 MG cells resulted in a significant inhibition of the ASIC1a currents evoked by the acidification of the extracellular solution to pH 5.5 (up to 11.8 ± 2.0% of the control, Figure 3d,e). This points on the same mambalgin-2 activity at the ASIC1a channels expressed in oocytes and U251 MG cells.

### 2.4. Mambalgin-2 Inhibits Growth of Glioma Cells via Interaction with ASIC1a

**Figure 4:**
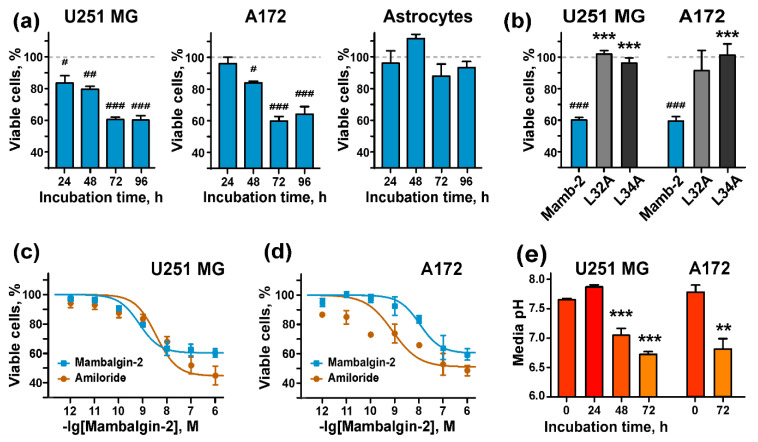
Mambalgin-2 influence on growth of U251 MG and A172 glioma cells and normal astrocytes: (**a**) 1 µM mambalgin-2 effect on U251 MG, A172 cells and astrocytes proliferation during different incubation times. Data are presented as % of control (untreated cells, dashed line) ± SEM (n = 4). # (*p* < 0.05), ## (*p* < 0.01), and ### (*p* < 0.001) indicate significant difference from the control according to One-way ANOVA followed by Dunnett’s test; (**b**) Influence of 1 μM mambalgin-2 and its Leu32Ala and Leu34Ala mutants on proliferation of U251 MG and A172 cells during 72-h incubation. Data are presented as % of control (untreated cells, dashed line) ± SEM (n = 4). ### (*p* < 0.001) indicates significant difference from the control according to One-way ANOVA followed Dunnett’s hoc test. *** (*p* < 0.001) indicates significant difference in activity of mutants and mambalgin-2 according to One-way ANOVA followed by Dunnett’s test; (**c**,**d**) effect of different mambalgin-2 and amiloride concentrations on U251 MG (**c**) and A172 (**d**) cell growth during 72-h incubation. The parameters describing concentration-effect curves (EC_50_, A_0_) are given in the Table 1. Data are presented as % of control (untreated cells) ± SEM (*n* = 3–11); (**e**) analysis of the pH value of culture medium during prolonged cultivation of U251 MG and A172 cells (*n* = 4). ** (*p* < 0.01) and *** (*p* < 0.001) indicate significant difference from the pH value of the culture media in an initial time point according two-tailed *t*-test.

ASIC1a channels participate in the regulation of the proliferation and migration of GBM cells [11]. Here, we tested the mambalgin-2 influence on U251 MG, A172, and normal astrocyte proliferation. The MTT viability test revealed that 1 µM mambalgin-2 reduced the proliferation of U251 MG and A172 cells, and this reduction became significant for both cell lines already after 48 h incubation (Figure 4a). The maximal inhibition effect (up to ~60% compared to the untreated cells) was reached upon 72-h incubation (Figure 4a). At the same time, mambalgin-2 did not affect the growth of normal astrocyte (Figure 4a).

The effect of mambalgin-2 on U251 MG and A172 cell viability upon 72-h incubation was concentration-dependent with EC_50_ of 10 ± 1.6 nM and 0.5 ± 0.2 nM, respectively (Figure 4c,d, Table 1). A comparison of the mambalgin-2 effect with action of amiloride revealed that the mambalgin-2 antiproliferative activity on A172 cells was weaker than the amiloride one, while on U251 MG cells, both amiloride and mambalgin-2 inhibited cell growth with comparable efficiencies (Figure 4c,d, Table 1).

To reveal a molecular target of mambalgin-2 in U251 MG and A172 cells, we used the variants of mambalgin-2 with mutations Leu32Ala and Leu34Ala with reduced inhibitory activity towards ASIC1a (Figure 2c, [31]). As was expected, both mutants did not influence on U251 MG and A172 cell proliferation (Figure 4b), pointing on ASIC1a as the primary target of mambalgin-2 in these malignant cells.

To study a relationship between the mambalgin-2 effect on glioma cell proliferation and cell cultivation time, we measured a pH value of the cell medium during cell growth. It was shown that the cultivation of U251 MG and A172 cells for a long time resulted in the significant acidification of cell media reaching the pH value ~6.7 upon 72-h cultivation, which is in line with the values determined for gliomas milieu [2,3].

To prove that mambalgin-2 can inhibit the ASIC1a channels at a pH close to that observed for the glioma environment, we measured the dose–response curve for the toxin at the channels expressed in *X. laevis* oocytes and activated at physiologically-relevant pH 6.6 (Figure 2b). The almost complete inhibition of the ASIC1a currents was achieved at 1 µM mambalgin-2 with IC_50_ 92 ± 17 nM.

Thus, the recombinant analogue of mambalgin-2 demonstrated the ASIC1a inhibitory activity at pH values corresponding to the brain tumor milieu [2,3], and its antiproliferative activity could correlate with the acidification of the cell medium.

### 2.5. Mambalgin-2 Induces Cell Cycle Arrest in U251 MG and A172 Cells

**Figure 5:**
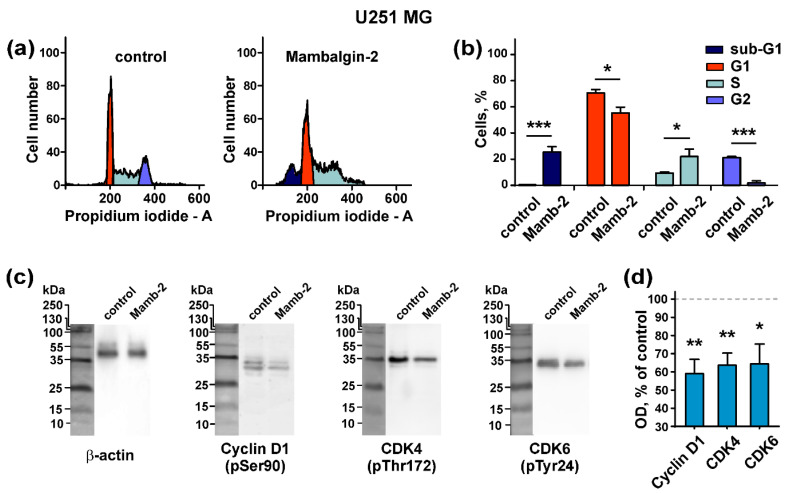
Mambalgin-2 influence on the cell cycle and activity of cell cycle regulators in U251 MG cells: (**a**) Representative nuclei population distributions of cells after 72-h incubation in absence (control) or presence of 1 µM mambalgin-2; (**b**) % of cells in each cell cycle phase determined by ModFitLT Software. Data are presented as % of cells in each cell cycle phase ± SEM, n = 4; * (*p* < 0.05) and *** (*p* < 0.001) indicate the significant difference from the control by two-tailed *t*-test; (**c**) Representative Western blots, showing the mambalgin-2 influence on phosphorylation of Cyclin D1 (pSer90), cyclin-dependent kinases (CDK)4 (pThr172), and CDK6 (pTyr24); (**d**) The optical density (OD) of blot bands for comparison of the Cyclin D1 (pSer90), CDK4 (pThr172), CDK6 (pTyr24) expression upon 72-h incubation of cells in absence (control) or presence of 1 µM mambalgin-2. Data are presented as normalized to the β-actin band intensity, where untreated cells are taken as the control (100%, dashed line) ± SEM, n = 6; * (*p* < 0.05) and ** (*p* < 0.01) indicate the significant difference between control and mambalgin-2 treated cells by two-tailed *t*-test.

It is known that PcTx1 and benzamil inhibit the proliferation of glioma cells and cause cell cycle arrest in G0/G1 phase with the simultaneous up-regulation of cyclin-dependent kinase inhibitors [11]. Here, we investigated the mambalgin-2 influence on the cell cycle progression in U251 MG and A172 cells. Flow cytometry analysis revealed that in U251 MG cells, mambalgin-2 induced a reduction of the cell number in the G1 and G2 cell cycle phases from ~71% to ~55% and from ~21% to ~2%, respectively, and increased the cell number in the S phase ~2.4-fold compared to untreated cells (control) (Figure 5a,b). These results point on the cell cycle arrest in the S phase in U251 MG cells upon the mambalgin-2 treatment. In addition, a significant increase in the sub-G1 population upon glioma cells incubation with mambalgin-2 was observed (from ~0% to ~25%) pointing on apoptosis induction (Figure 5a,b).

**Figure 6:**
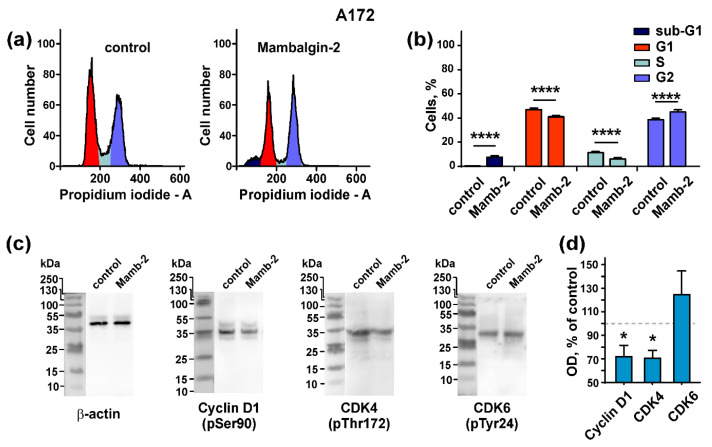
Mambalgin-2 influence on the cell cycle and activity of cell cycle regulators in A172 cells: (**a**) Representative nuclei population distributions of cells after 72-h incubation in absence (control) or presence of 1 µM mambalgin-2; (**b**) % of cells in each cell cycle phase determined by ModFitLT Software. Data are presented as % of cells in each cell cycle phase ± SEM, n = 4; **** (*p* < 0.0001) indicates the significant difference from the control by two-tailed *t*-test; (**c**) Representative Western blots, showing the mambalgin-2 influence on phosphorylation of Cyclin D1 (pSer90), CDK4 (pThr172), and CDK6 (pTyr24); (**d**) The optical density (OD) of blot bands for comparison of the Cyclin D1 (pSer90), CDK4 (pThr172), CDK6 (pTyr24) expression upon 72-h incubation of the cells in absence (control) or presence of 1 µM mambalgin-2. Data are presented as normalized to the β-actin band intensity, where untreated cells are taken as the control (100%, dashed line) ± SEM, n = 6; * (*p* < 0.05) indicates the significant difference between the control and mambalgin-2 treated cells by two-tailed *t*-test.

In A172 cells, the percentage of cells in the G1 and S cell cycle phases was significantly reduced upon mambalgin-2 treatment (from ~48% to ~41% and from ~11% to ~5%, respectively). Contrarily, the number of cells in the G2 phase was significantly increased upon the mambalgin-2 treatment (from ~38% to ~47%), pointing on the cell cycle arrest in the G2/M phase. Similar to U251 MG cells, an increase in the sub-G1 cell population upon incubation with mambalgin-2 (from ~0% to ~7%) was observed (Figure 6a,b).

To further study the mambalgin-2 influence on the cell cycle, we analyzed the toxin’s action on phosphorylation of cell cycle regulators, such as cyclin D1 and cyclin-dependent kinases 4 and 6 (CDK4 and CDK6). Western blotting showed that 72-h incubation of U251 MG cells with 1 µM mambalgin-2 significantly inhibited the phosphorylation of cyclin D1 and cyclin-dependent kinases CDK4 and CDK6 (Figure 5c,d; Figure S2). The incubation of A172 cells with 1 µM mambalgin-2 during the 72 h led to the inhibition of cyclin D1 and CDK4 phosphorylation, but not of CDK6 (Figure 6c,d; Figure S3).

Thus, the incubation of U251 MG and A172 cells with mambalgin-2 induces cell cycle arrest in the S-phase and G2/M phase, respectively, and inhibits the phosphorylation of the cell cycle regulators.

### 2.6. Mambalgin-2 Induces Apoptosis in U251 MG and A172 Cells

**Figure 7:**
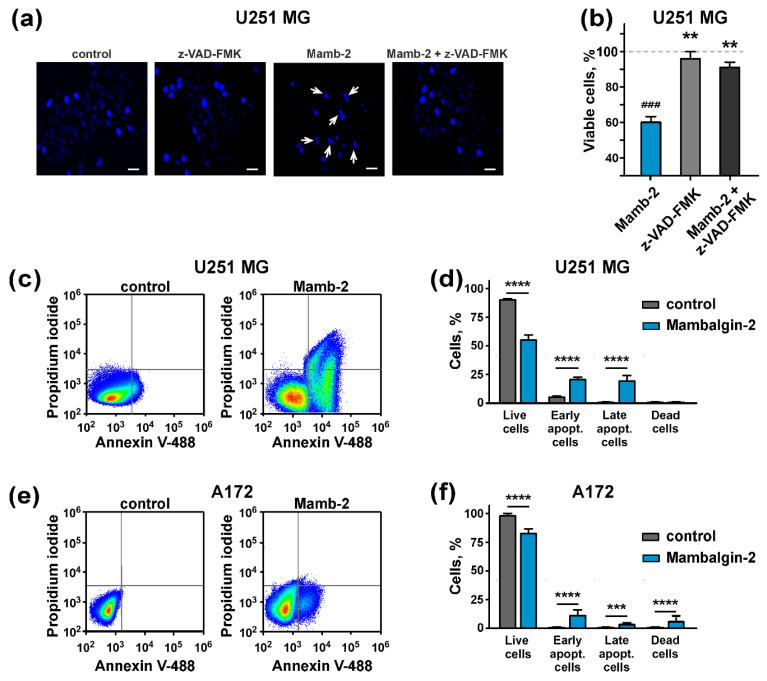
Apoptosis induction by mambalgin-2 in U251 MG and A172 cells: (**a**) Microscopic examination of nuclei morphology of U251 MG cells after their incubation with 1 µM mambalgin-2 in absence or presence of pan-caspase inhibitor z-VAD-FMK, scale bar = 10 µm; (**b**) Influence of mambalgin-2 and z-VAD-FMK on U251 MG cell proliferation. Data are presented as % of control (untreated cells, dashed line) ± SEM (n = 4). ### (*p* < 0.001) indicates significant difference between treated and untreated cells according to One-way ANOVA followed by Dunnett’s test. ** (*p* < 0.01) indicates significant difference from the mambalgin-2 treated cells according to One-way ANOVA followed by Dunnett’s test; (**c**,**e**) representative pictures of phosphatidylserine externalization analysis upon the mambalgin-2 treatment of U251 MG and A172 cells by flow cytometry with Annexin V-488 and Propidium iodide; (**d**,**f**) percentage of U251 MG and A172 cells with externalized phosphatidylserine and bound propidium iodide upon 72-h incubation in absence (control) or presence of 1 µM mambalgin-2. The data are presented as % of live, early apoptotic, late apoptotic and dead cells ± SEM (n = 4). *** (*p* < 0.001) and **** (*p* < 0.0001) indicate the significant difference of the groups from each other by a two-tailed *t*-test.

The formation of the sub-G1 peak on the cell cycle histograms (Figure 5; Figure 6) is characteristic for apoptosis [33]. In line with this, the microscopic examination revealed nuclei fragmentation of U251 MG cells after 72-h incubation with 1 µM mambalgin-2, pointing on apoptosis induction (Figure 7a). To prove apoptosis, we used the pan-caspase inhibitor z-VAD-FMK, which irreversibly blocks caspases without a cytotoxic effect. The application of z-VAD-FMK completely cancelled nuclei fragmentation upon the mambalgin-2 treatment (Figure 7a), and abolished the mambalgin-2 antiproliferative effect on U251 MG cells (Figure 7b).

To further justify apoptosis induction in U251 MG and A172 cells upon the mambalgin-2 treatment, we used Annexin V/Propidium iodide assay. The analysis by flow cytometry revealed that the number of U251 MG cells with the externalized phosphatidylserine increased upon the 72-h incubation with 1 μM mambalgin-2 from ~2.3% to ~22%. Moreover, ~19% of U251 MG cells possess not only externalized phosphatidylserine, but also bind propidium iodide upon the mambalgin-2 treatment (Figure 7c,d). This points on a membrane integrity loss and late apoptosis induction, which is consistent with an appearance of the sub-G1 cell population (Figure 5a,b). The number of A172 cells with the externalized phosphatidylserine increased upon the 72-h treatment with 1 μM mambalgin-2 from ~0% to ~9% (Figure 7e,f). Similar to U251 MG cells, incubation with mambalgin-2 resulted in an increase of the A172 cell population with externalized phosphatidylserine and simultaneously bound propidium iodide from ~0% to 2%. Moreover, mambalgin-2 enhances the number of dead A172 cells from ~1% to 5% (Figure 7e,f).

### 2.7. Mambalgin-2 Inhibits Growth of Primary Cells Obtained from GBM Patient

**Figure 8:**
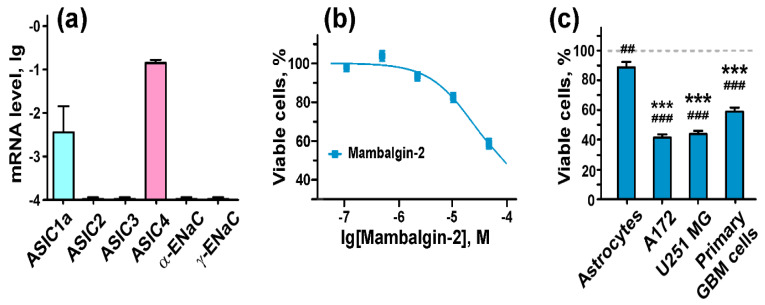
Mambalgin-2 effect on cells of primary culture obtained from a patient with glioblastoma (GBM). (**a**) Analysis of ASICs and ENaCs gene expression in primary GBM cells by qPCR. Gene expression was normalized to the β-*ACTIN*, *GPDH* and *RPL13a* housekeeping genes and presented as lg of relative mRNA level ± SEM (*n* = 3–5); (**b**) Influence of different mambalgin-2 concentrations on viability of primary GBM cells. Cells were incubated for five days with mambalgin-2 and their viability was accessed by Alamar Blue assay. Data are presented as % of control (untreated cells) ± SEM (n = 3); (**c**) Comparison of the 50 µM mambalgin-2 inhibitory effect on viability of normal astrocytes, U251 MG, A172, and primary GBM cells by Alamar Blue assay. Data are presented as % of control (untreated cells, dashed line) ± SEM (n = 3). ## (*p* < 0.01) and ### (*p* < 0.001) indicate significant difference between treated and untreated cells according to One-way ANOVA followed by Dunnett’s test. *** (*p* < 0.001) indicates significant difference of mambalgin-2 action on cells from the effect on astrocytes according to One-way ANOVA followed by Dunnett’s test.

To evaluate the physiological relevance of our data, we tested the mambalgin-2 antiproliferative activity on low-passage cells (neurospheres) from a patient with GBM [34]. First, we showed that primary GBM cells express the *ASIC1a* and *ASIC4* genes, but not *ASIC2, ASIC3*, α- or γ-*ENAC* mRNA (Figure 8a). The alamar blue viability test revealed that mambalgin-2 inhibited the growth of primary GBM cells after five-day incubation in the dose–dependent manner (Figure 8b). A comparative analysis revealed that 50 µM mambalgin-2 inhibited the growth of high-passage U251 MG and A172 cells to ~40% and 42%, respectively, and low-passage primary GBM cells to ~60% compared to untreated cells upon the five-day treatment in the same conditions (Figure 8c). Notably, 50 µM mambalgin-2 inhibited the growth of normal astrocytes to ~90%, which is significantly lower than the toxin’s antiproliferative effect on glioma model cells and primary GBM cells (Figure 8c).

## 3. Discussion

GBM is the most common and aggressive of the primary brain tumors. Currently used methods of treatment have very limited therapeutic effect, especially on the late stages of the disease. Due to the poor outcome of GBM therapy by DNA-alkylating agents, the targeting of biomolecules implicated in the GBM progression is considered as a perspective GBM therapy strategy. To date, different inhibitors of the PI3K/mTOR pathway, hepatocyte growth factor receptor (MET gene), fibroblast, epidermal, and vascular endothelial growth factors receptors (FGFR, EGFR, and VEGFR, respectively) are being investigated for GBM treatment, but only VEGF antibody bevacizumab improved the progression-free survival of GBM patients [35]. Recently, it was demonstrated that the modulation of the nicotinic acetylcholine receptors (nAChRs) also could be implicated in the control of glioma cell growth. Thus, human secreted protein SLURP-1 inhibited the proliferation of A172 and U251 MG cells with nanomolar activity via interaction with nAChRs [36]. Another modulator of nAChRs, Lynx1, was demonstrated to induce cell cycle arrest and apoptosis in lung carcinoma cells [37], pointing on its possible usage for glioma treatment too. Thus, the search for new molecular targets for GBM treatment is ongoing.

One of the differences between malignant glioblastoma cells and native astrocytes is the presence of amiloride-sensitive sodium conductance that is attributed to ASICs or their complex with EnaCs [1]. This amiloride-sensitive sodium conductance is presented in high-grade (grades III and IV) tumors and is not observed in normal astrocytes or low-grade gliomas. ASICs and EnaC channels are known to participate in the regulation of many essential processes in GBM cells [19,20], and in malignant and normal astrocytes [19,20,38]. The activation of these channels leads to the inhibition of apoptosis and stimulates the proliferation, migration and invasion of glioma and carcinoma cells, and contrary, their inhibition leads to suppression of tumor growth [19]. Moreover, the malignant transformation of astrocytes leads to changes in the expression pattern of ASIC subunits. These observations allow us to consider the ASIC/ENaC channels and their inhibitors as promising targets for GBM treatment.

Here, we showed that only glioma cells express mRNA of the ASIC1a subunit, while no gene expression of this type of the channels was found in normal astrocytes (Figure 1 and Figure 8a). Notably, the gene expression of other ASIC and EnaC subunits was similar both in model malignant and normal cells, except ASIC3, in which expression was higher in normal astrocytes (Figure 1). Analysis of the TCGA GBM database (TCGA GBM and GTEX studies via Xena platform) revealed that the ASIC1 mRNA expression is up-regulated in tissue samples of patients with glioma compared to healthy people, while ASIC2 and ASIC3 gene expression is down-regulated [13], consistent with our data obtained on primary GBM cells (Figure 8a). Astrocyte activation by lipopolysaccharides (LPS) is known to increase the level of ASIC1a [39], and we can suppose that the ASIC1a expression increase in tumor cells is linked to inflammatory signaling activation in malignant cells.

The viability test demonstrated that mambalgin-2 effectively suppresses proliferation of A172 glioblastoma and U251 MG glioma cells with EC_50_ in nanomolar range (1–10 nM). The maximal inhibitory effect ~60% was observed at 1 µM mambalgin-2 (Figure 4c,d, Table 1). Moreover, the inhibitory activity of mambalgin-2 was confirmed on primary GBM cells obtained from a patient (Figure 8b), and its magnitude is comparable with the toxin’s activity on model glioma cells (Figure 8c). Notably, no inhibitory activity of 1 µM mambalgin-2 and only 10% growth inhibition for 50 µM mambalgin-2 was found on normal astrocytes (Figure 4a and Figure 8c). This points to mambalgin-2 as a selective agent for GBM treatment.

Previously, other ASIC1a inhibitors such as amiloride, benzamil, and PcTx1 were also shown to inhibit proliferation of glioma cells. Thus, 100 nM PcTx1 and 100 µM benzamil inhibited D54-MG cell proliferation upon 24 h incubation by ~30% and ~40%, respectively [11], while 100 µM and 500 µM amiloride inhibited growth of U118 glioma cell upon 48 h treatment by ~40% and ~75%, respectively [21]. Here, we demonstrated that 1 µM amiloride inhibits the growth of A172 and U251 MG cells up to ~45% with EC_50_ ~0.8 and 3.6 nM, respectively (Figure 4c,d, Table 1). Nevertheless, amiloride and PcTx1 have serious limitations for glioma therapy. Indeed, amiloride demonstrates low selectivity on ASICs, inhibiting ASIC1a, ASIC1b, ASIC2a, and activating homomeric ASIC3 and heteromeric ASIC3/ASIC1b-channels at a neutral pH [40,41]. PcTx1 inhibits ASIC1a [42], ASIC1a/2b [43], and ASIC1a/2a [44], and activates the ASIC1b isoform [45] and ASIC1a/ASIC2a heteromeric channels [46]. In contrast, in the central nervous system, mambalgin-2 selectively inhibits only homo and heteroreceptors containing ASIC1a [27]. Thus, ASIC1a could be considered a marker of cell sensitivity to mambalgin-2. Notably, GBM stem cells, which are more malignant and resistant to chemotherapy, also express functional ASIC1a [47], therefore mambalgin-2 could inhibit proliferation of GBM stem cells too, thus increasing efficiency of antitumor therapy.

Electrophysiology experiments on *Xenopus laevis* oocytes demonstrated that mambalgin-2 effectively blocks currents at ASIC1a activated not only at pH 5.5, as was reported earlier [26], but also at a physiologically relevant pH of 6.6 (Figure 2c). Biosensor Imaging of Redundant Deviation in Shifts (BIRDS) revealed the range of glioblastoma extracellular pH 6.5–7.1 (mean 6.83 ± 0.15) in the rat brain bearing a U251 tumor [48], in line with microelectrode measurements in patient samples [2,3]. ASIC1a channels are activated by a pH drop below 6.9 [30], and probably the acidification associated with a glioma cell density increase and active metabolism of malignant cells results in the activation of the ASIC1a channels in gliomas/glioblastomas. Notably, the life-time of the ASIC1a channels in the open state is very short, and in hundreds of milliseconds the channels switch from the low-pH open to the low-pH desensitized state [49]. The mambalgins interact with ASICs in the desensitized state and prevents further activation of the channel [27,28]. Our data suggest that mambalgin-2 inhibits the growth of glioma cells by an interaction with ASIC1a in the desensitized state, and the magnitude of the toxin’s antiproliferative effect correlates with a pH value of the cell environment (Figure 4a,e). Thus, pH sensors such as the ASIC1a channels expressed in glioma cells for adaptation to the acidic environment could become perspective targets for glioma treatment.

Cell cycle arrest is mediated by the cell cycle regulators: cyclins and cyclin-dependent kinases (CDKs) [50]. Cyclin D1 forms complexes with the cyclin-dependent kinases CDK4 and CDK6, in which CDKs undergo activation by phosphorylation [51]. After activation, complexes of Cyclin D1/CDK phosphorylate the retinoblastoma protein (Rb protein, a DNA synthesis inhibitor), resulting in the Rb protein inactivation and the entering of cells into the S phase of the cell cycle [50]. In glioblastomas, the Rb protein is usually inhibited either by a *Rb* gene deletion or by *CDK4* and *CDK6* gene amplification [52], thus promoting tumor cell proliferation. Therefore, the inhibition of the cyclin D1 and CDK activity could prevent the Rb protein inactivation and lead to the cell cycle arrest. Presently, this approach is considered one of the perspective strategies for GBM treatment [52]. Our results show that mambalgin-2 reduces the phosphorylation of Cyclin D1 and CDKs in U251 MG and A172 cells (Figure 5c,d and Figure 6c,d), pointing on the inactivation of the Cyclin D1/CDK complexes, and therefore on a possible restoration of the Rb protein activity and inhibition of DNA synthesis. We suppose that a decrease of the Cyclin D1, CDK4 and CDK6 phosphorylation in U251 MG cells upon the mambalgin-2 treatment may induce the cell cycle arrest both in the G1 and S phases, but the cell number in the G1 phase may be decreased due to apoptosis induction (Figure 7). The subsequent appearance of the sub-G1 peak, which is formed by the destructed nuclei of cells initially arrested in the G1 cell cycle phase, favors this assumption (Figure 5a). In line with this, the decrease of the Cyclin D1, CDK4, and CDK6 phosphorylation can be observed in the cell cycle arrest both in the G1 and S phases [53,54,55]. In A172 cells, we observed a slightly different situation: mambalgin-2 does not influence the phosphorylation of CDK6 and causes the cell cycle arrest in the G2/M phase (Figure 6). This difference could be explained by different genotypes of U251 MG and A172 cells and as a result of the different gene expression in cell subpopulations in each cell cycle phase, as it was reported early for liposarcoma of breast cancer cells [56]. This assumption is indirectly supported by an initially lower level of the cell population in the G2 cell cycle phase in untreated U251 MG cells in comparison with untreated A172 cells (Figure 5b and Figure 6b).

Thus, we propose the following mechanism of mambalgin-2 action: interaction with ASIC1a in U251 MG and A172 cells affects the proliferation-related intracellular pathways, which results in the down-regulation of the phosphorylation of cyclin D1 and cyclin-dependent kinases. This inhibits DNA synthesis by restoring of the Rb protein in the active state and leads to the cell cycle arrest in the G1 and S or G2/M phases in U251 MG and A172 cells, respectively. In parallel, apoptosis occurs in both cell lines, but in A172 cells this process proceeds in a milder form than in U251 MG cells, and the membrane integrity loss is not manifested upon 72 h of incubation with mambalgin-2. In U251 MG cells, apoptosis after 72-h incubation with mambalgin-2 is more pronounced; the cell population at the late apoptosis stage appears, and nuclei of these cells form the sub-G1 peak on the cell cycle histogram (Figure 5b). Altogether, these events lead to the inhibition of glioma cell proliferation.

The promising results obtained here, including that obtained on primary GBM cells, allow us to consider mambalgin-2 a useful hit for the design of novel therapy strategies for GBM treatment. Nevertheless, a question about the permeability of the blood–brain barrier (BBB) for mambalgin-2 remains open. Recently, we showed that water-soluble domain of the nAChR modulator ws-Lynx1 crosses BBB in mice, despite the quite high molar mass of the protein (~8 kDa) [57]. We proposed that ws-Lynx1 can penetrate BBB by transcytosis via interaction with nAChRs expressed on endothelial cells [57]. Besides nAChRs, human brain endothelial cells express ASIC1a [58], so we suppose that mambalgin-2 can also pass through BBB by transcytosis, however further investigation of this issue is required. On the other hand, BBB is known to be damaged in GBM due to the down-regulation of the expression of intercellular junctions and astrocyte end feet displacement, and the barrier function of the brain endothelium is impaired [59]. Thus, the ability of different drugs to cross BBB is increased in GBM. Moreover, peptide variants or chemical mimetics designed on the basis of mambalgin-2 could demonstrate an enhanced ability to cross BBB and may be more preferable for clinical use.

## 4. Materials and Methods

### 4.1. Recombinant Mambalgin-2 Production

Recombinant mambalgin-2 and its two mutant variants with Leu32Ala and Leu34Ala substitutions were produced in *E. coli* as described previously [29]. Briefly, washed inclusion bodies were dissolved in buffer containing 8 M urea, 0.4 M sodium sulfite, 0.15 M sodium tetrathionate and left overnight under stirring. Sulfited mambalgin-2 was purified on DEAP-sferonit-OH ion exchange resin (joint development by the Institute of Highly Pure Biopreparations, St. Petersburg, and the Institute of Bioorganic Chemistry, Russia). Sulfited protein was reduced by excess of DTT and additionally purified by HPLC (Jupiter C4, A300, 10 × 250 mm, Phenomenex, 20–45% gradient of acetonitrile in presence of 0.1% TFA for 40 min). Sample was lyophilized and dissolved in degassed refolding buffer containing 50 mM Tris-HCl, 1.5 M urea, 0.5 M L-arginine, pH 8.0 in presence of reduced (GSH) and oxidized (GSSG) glutathione (molar ratio: 1:50:500 mambalgin-2/GSSG/GSH at a protein concentration of 0.01 mg/mL). Refolding was performed at 4 °C for 72 h. Refolded mambalgin-2 was analyzed and purified by HPLC (Jupiter C4, A300, 10 × 250 mm, Phenomenex, 20–45% gradient of acetonitrile in presence of 0.1% TFA for 40 min.) The purity and homogeneity of the refolded mambalgin-2 (>95%) were confirmed by HPLC, MALDI-MS, and SDS-PAGE. Disulfide bond formation was confirmed in the reaction with Ellman’s reagent (Sigma-Aldrich, Louis, MO, USA). The correct spatial structure was confirmed by 1D ^1^H NMR-spectroscopy (Figure S1).

### 4.2. Real-Time PCR

mRNA was isolated with ExtractRNA reagent (Evrogen, Moscow, Russia), treated by DNAse I (Sigma-Aldrich) and purified with CleanRNA Stanadart kit (Evrogen). cDNA was synthesized by Mint reverse transcriptase kit (Evrogen). After that, qPCR was performed with ready to use SYBRGreen HS mix (Evrogen) and primers specific to the ASIC1a, ASIC2, ASIC3, ASIC4, α-ENaC and γ-ENaC genes (Table S1) on Roche LightCycler 96 amplifier (Roche, Basel Switzerland). Data were analyzed by ∆∆Ct method on LightCycler SW software (Roche) and a gene expression was normalized to the *β-ACTIN*, *GPDH* and *RPL13a* housekeeping genes.

### 4.3. Electrophysiological Recordings in Xenopus oocytes

The research was carried out in an AAALAC-accredited organization according to the standards of the Guide for Care and Use of Laboratory Animals (8th edition, Institute for Laboratory Research of Animals). All experiments with *Xenopus laevis* female frogs were approved by the IBCh RAS IACUC (Protocol Number 267/2018; date of approval: 28 February 2019). *X. laevis* oocytes were removed surgically under anesthesia (0.17% solution of tricane methanesulfonate), defolliculated, and injected with 2.5–10 ng of capped RNA. Capped RNA transcripts were synthesized from linearized cDNA templates using a T7 RiboMAX^TM^ large-scale RNA production system (Promega, Madison, WI, USA) according to a protocol for capped transcripts supplied by the manufacturer from linearized PCi plasmid containing rat ASIC1a. After injection, the oocytes were kept for 2–3 days at 19 °C and then up to 7 days at 15 °C in a ND-96 medium containing 96 mM NaCl, 2 mM KCl, 1.8 mM CaCl_2_, 1 mM MgCl_2_, 5 mM HEPES (pH = 7.4) and gentamycin (50 g/mL). Two-electrode voltage clamp recordings were performed using a GeneClamp 500 amplifier (Axon Instruments, San Jose, CA, USA), and data were filtered at 20 Hz and digitized at 100 Hz by an AD converter L780 (L-Card, Moscow, Russia) using homemade software. Microelectrodes were filled with 3 M KCl solution. An external ND96 solution with pH 7.4 was used. To induce specific currents, we employed ND-96 modified solutions (in which 5 mM HEPES was substituted to 5 mM MES (pH 5.5) or 10 mM MOPS (pH 6.6)). All solutions of testing compounds were supplemented with 0.1% BSA. A computer-controlled valve system for fast solution application was used to switch solutions. The acid currents were evoked by a 1 s pH drop from the base value of 7.4 to 5.5 or 6.6 in the external oocyte solution. Recombinant mambalgin-2 or its mutants were applied for 15 s before and during the activation impulse to prevent non-specific adsorption. Data analysis was performed using OriginPro 8.6 software (OriginLab, Farmington, ME, USA).

The dose–response curve was fitted using the four-parameter equation: F(x) = ((a1 − a2)/(1 + (x/x0)ˆn_H_)) + a2, where F(x) is the response value at given concentration of mambalgin-2; a1 is the control response value; a2 is the response value at maximal inhibition (% of the control); x is the concentration of mambalgin-2; x0 is the IC_50_ value; and n_H_ is the Hill coefficient. Data analysis was performed using OriginPro 8.6 software (OriginLab).

### 4.4. Patch Clamp on U251 MG Cells

Patch-clamp experiments on U251 MG cells were performed in the whole-cell configuration. Currents were recorded using a patch-clamp amplifier (Axopatch 200 B), the Analog-Digital Interface (Digidata 1550 A) and Clampex software (Molecular Devices, USA). Patch pipettes were made from borosilicate glass at a P-97 puller (Sutter Instrument, USA) and had a resistance of 3–6 MΩ, when filled with an intracellular solution containing 140 mM K-Aspartate, 5 mM NaCl, 2 mM EGTA/KOH, 1 mM MgCl_2_, 20 mM HEPES/TrisOH and 0.176 mM CaCl_2_ to establish free ionized calcium concentration [Ca^2+^] i at 0.01 µM. The bath extracellular solution contained 145 mM NaCl, 2 mM CaCl_2_, 1 mM MgCl_2_, and 10 mM HEPES/Tris (pH = 7.4). For bath solution with pH 5.5, HEPES was replaced by MES (pH = 5.5). Membrane voltage was clamped to −50 mV. It should be noted that we were unable to register the effects of different reagents in one experiment because of unstable patches that could not withstand more than three or four solution changes. Data were filtered at 200 Hz with low-pass filter, and analyzed using pClamp software. Benzamil was from Sigma-Aldrich.

### 4.5. Cell Cultivation and Proliferation Assay

Human glioma U251 MG cells (Russian Cell Culture Collection, Institute of Cytology RAS, Novosibirsk Oblast, Russia) were grown in Iscove’s Modified DME medium (IMDM) with phenol red (PanEco, Moscow, Russia), 10% fetal calf serum (GE Healthcare, Waukesha, WI, USA), 2 mM L-glutamine (PanEco). Human glioma A172 (Institute of Cytology RAS) were grown in RPMI-1640 media, supplemented with 10% fetal calf serum. Human astrocytes (Cell Applications, San Diego, CA, USA) were grown in Neurobasal A medium (Thermo Fisher, Waltham, MA, USA) with addition of G-5 (PanEco) Cells were maintained at 37 °C in a humidified atmosphere with 5% CO_2_. All types of cells were subcultured twice per week.

To study the mambalgin-2 influence on a cell proliferation, the cells were seeded in 96-well cell culture plates (0.5 × 10^4^ cells/well) and grown for 24 h. Thereafter, mambalgin-2 (from the 1 mM 100% DMSO stock solution) was dissolved in a cell medium and added to the cells at concentrations from 10^−^^13^ to 10^−^^6^ M for further incubation during the 72 h. Every 24 h cell media was aspirated and replaced by fresh one, containing pre-dissolved mambalgin-2 in the same concentration as in initial media. The maximal DMSO concentration did not exceed 0.1%. The added DMSO did not influence a cell growth as was established in additional experiments.

The cell viability was characterized using the MTT test. MTT reagent was dissolved in PBS and added to plate wells (0.1 mg per well) and cells were incubated with MTT for 2 h in humidified atmosphere. After that, formazan crystals were dissolved in acidic isopropanol and cell viability was evaluated spectrophotometrically by measuring the absorbance on microplate reader (Bio-Rad 680, Bio-Rad, Hercules, CA, USA) at 540 nm with background subtraction at 655 nm. For comparison of mambalgin-2 action on A172, U251 MG cells, astrocytes and primary glioma cultures cell viability was analyzed by Alamar Blue (see below). Data was analyzed and fitted by Graphpad Prism 6.0 software (GraphPad Software, San Diego, CA, USA).

For investigation of apoptosis in U251 MG cells, cells were incubated with 10 μM of z-VAD-FMK pan-caspase inhibitor (Santa-Cruz, CA, USA) for 30 min. Then, U251 MG cells were rinsed twice with a fresh medium, 1 µM mambalgin-2 and/or 10 μM z-VAD-FMK were added to the cells, and they were cultured further for 72 h. Every 24 h cell media was aspirated and replaced by fresh one, containing pre-dissolved mambalgin-2 and/or z-VAD-FMK in the same concentration as in initial media.

### 4.6. Primary Cell Culture from a GBM Patient

Glioma tissue sample was obtained from N.N. Burdenko National Medical Research Center of Neurosurgery (Moscow, Russia) and processed to the research laboratory after de-identification of the sample. Diagnosis was confirmed by morphological studies. The study was approved by the ethics committees of the N.N. Burdenko National Medical Research Center of Neurosurgery. The use of the de-identified tissue was exempt from requiring consent. Primary culture of GBM cells as neurospheres was established as described previously [35]. Briefly, GBM specimens were collected during surgery under preoperative MRI-guided navigation and mechanically dissociated into pieces with 1–3 mm diameter. The samples were then treated with trypsin for 20 min at +37 °C to obtain single cells. Cell suspensions were run through Lympholyte-H separation (Cedarlane Labs, Canada) to remove Red Blood Cells and debris according to manufacturer’s specifications. Established cell line was cultivated for no longer than 10 passages in DMEM/F12 medium containing 2% B27 supplement (Thermo Fisher), 1% Penicillin–Streptomycin solution (Thermo Fisher), 2.5 μg/mL heparin (Sigma-Aldrich), 20 ng/mL basic fibroblast growth factor (bFGF; Sigma-Aldrich), and 20 ng/mL epidermal growth factor (EGF; Sigma-Aldrich). EGF and bFGF were added twice a week and the cultural medium was changed every 7 days.

Viability of low-passage primary GBM cells and comparative analysis of the mambalgin-2 effect on high-passage U251 MG and A172 cells, low-passage primary GBM cells, and normal astrocytes was evaluated by AlamarBlue reagent (DAL1100, Thermo Fisher). Cells were seeded at 6 × 10^3^ cells per well in a 96 well plate. Next day mambalgin-2 was added. After 5-day incubation AlamarBlue reagent was added into each well and 4 h later fluorescence was measured (Excitation 515–565 nm, Emission 570–610 nm) using a Synergy HTX multi-mode reader (BioTek, Winusky, VT, USA).

### 4.7. Cell Cycle Analysis

Cells we seeded in 6-well culture plates (12.5 × 10^3^ cells per well) and incubated with 1 μM mambalgin-2 for 72 h with media aspiration every 24 h. Then the cells were detached from the wells by trypsin-EDTA, washed with Earl balanced salt solution (EBSS), and fixed in ice-cold 70% ethanol for 12 h. After fixation, the cells were washed twice by EBSS, and DNA was extracted by 5 min incubation with the DNA extraction buffer (200 mM Na_2_HPO_4_ with 0.004% Triton X-100, pH = 7.8). Then the cells were washed with EBSS, resuspended in DNA staining solution (EBSS, 50 mg/mL propidium iodide, 0.2 mg/mL DNAse free RNAse A), and analyzed by FACSCalibur flow cytometer (Becton Dickinson, Becton Drive Franklin Lakes, NJ, USA). The data were analyzed using ModFit LT software (Verity Software, Topsham, ME, USA).

### 4.8. Western Blotting

Cells were lysed in RIPA buffer with SIGMAFAST protease inhibitor cocktail (Sigma-Aldrich), lysates were diluted in the loading buffer (120 mM Tris-HCl, 20% [*v*/*v*] glycerol, 10% [*v*/*v*] mercaptoethanol, 4% [*w*/*v*] sodium dodecyl sulfate, and 0.05% [*w*/*v*] bromophenol blue, pH 6.8), submitted to gel electrophoresis, blotted onto nitrocellulose membranes (Santa-Cruz) and blocked in 5% skim-milk (Dia-m, Moscow, Russia) for 2 h. The membranes were incubated overnight at 4 °C with primary rabbit antibodies against Cyclin D1 (pSer90, Antibodies-online, ABIN6271254), CDK4 (pThr172, Antibodies-online, ABIN6271182), CDK6 (pTyr24, Antibodies-online, ABIN319289), or mouse anti-β-actin antibody (R&D, MAB8929); washed 3 times with TBS (50 mM Tris, 150 mM NaCl, pH = 7.4) + 0,1% Tween-20 (Applichem, Darmstadt, Germany) and incubated with HRP-conjugated secondary anti-rabbit antibody (Jackson Immunoresearch, West Grove, PA, USA 111-035-003) or anti-mouse antibody (Jackson Immunoresearch, 715-035-150) for 1 h (20 °C). After that, membranes were washed 4 times with TBS + 0.1% Tween-20, and an HRP signal was detected by ECL substrate (Bio-Rad, Hercules, CA, USA) with a VersaDoc 4000 chemidocumenter (Bio-Rad) and quantified using “gel analyzer” option of ImageJ software (NIH, Bethesda, MD, USA).

### 4.9. Fluorescent Microscopy

U251 MG cells were seeded in 96-well cell culture plate and incubated with 1 µM mambalgin-2 and/or 10 μM z-VAD-FMK for 72 h as previously described. After that cell nuclei were stained with 1 μM of Hoechst 33,342 (PanEco) and observed under 20x objective of Nikon TS-100 (Nikon, Tokyo, Japan) fluorescent microscope.

### 4.10. Analysis of Phosphatidylserine Externalization

To investigate apoptosis in U251 MG and A172 cells, we used Annexin V for detection of the phosphatidylserine externalization, one of the early apoptosis markers. Briefly, cells were seeded on a 35-mm Petri dish (1 × 10^5^ cells/dish) and incubated with 1 μM of mambalgin-2 for 72 h as described above. After incubation, the cells were detached by the Versene solution and washed in annexin-binding buffer (V13246, Thermo Fisher Scientific, Waltham, MA, USA). Then, the cells were incubated with Annexin V conjugated to Alexa 488 (A13201 Thermo Fisher Scientific) for 20 min, washed by annexin-binding buffer and were analyzed on ACEA Novocyte flow cytometer (ACEA Biosciences, San Diego, CA, USA). The data were analyzed using NovoExpress software.

## 5. Conclusions

We demonstrated the selective expression of the ASIC1a channels in glioma model cells and primary GBM cells, but not in normal astrocytes. We obtained the recombinant analog of mambalgin-2 from *Dendroaspis polylepis* and showed that it inhibits amiloride-sensitive currents in ASIC1a expressed in *Xenopus* oocytes and U251 MG glioma cells. In line with the absence of ASIC1a in normal astrocytes, mambalgin-2 inhibits the growth of glioma cells and primary GBM cells, but not of normal astrocytes, and also down-regulates phosphorylation of the cell cycle regulators and induces the cell cycle arrest and apoptosis in U251 MG and A172 cells. Mambalgin-2 can be considered a perspective prototype for new drugs not only for glioma treatment, but other cancers with selective ASIC1a overexpression.

## Figures and Tables

**Table 1:** Parameters descripting the mambalgin-2 effect on U251 MG and A172 cells proliferation.

Cells	Amiloride		Mambalgin-2	
	A_0_, %	EC_50_, nM	A_0_, %	EC_50_, nM
U251 MG	44.84 ± 4.0	3.6 ± 0.2	61.78 ± 1.4	0.5 ± 0.2
A172	48.06 ± 4.6	0.8 ± 0.02	60.15 ± 3.0	10 ± 1.6

## Supplementary Materials

The following are available online at https://www.mdpi.com/2072-6694/12/7/1837/s1, Figure S1: Characterization of the refolded mambalgin-2; Figure S2: Western blots from 6 independent portions of U251MG cells, showing the mambalgin-2 influence on phosphorylation of Cyclin D1 (pSer90), CDK4 (pThr172), and CDK6 (pTyr24); Figure S3: Western blots from 6 independent portions of A172 cells, showing the mambalgin-2 influence on phosphorylation of Cyclin D1 (pSer90), CDK4 (pThr172), and CDK6 (pTyr24). Table S1: Primers, used for qPCR.
