# Supplementary material for: Mambalgin-2 Induces Cell Cycle Arrest and Apoptosis in Glioma Cells via Interaction with ASIC1a
Source: Cancers (Basel). 2020 Jul 8;12(7):1837. doi: 10.3390/cancers12071837 (PMC7408772; doi:10.3390/cancers12071837)
Supplement: Supplementary file 1 [file cancers-12-01837-s001.pdf]

cancers

MDPI

Article

Mambalgin-2 induces cell cycle arrest and apoptosis in glioma cells via interaction with ASIC1a

Supplementary material:

Maxim Bychkov, Mikhail Shulepko, Dmitry Osmakov, Yaroslav Andreev, Anastasia Sudarikova, Valeria Vasileva, Marat S. Pavlyukov, Yaroslav Latyshev, Alexander A. Potapov[4], Mikhail Kirpichnikov, Zakhar Shenkarev, Ekaterina Lyukmanova

Table S1. Primers, used for qPCR.

Gene	Primer		Amplicon Size, bp
	Forward	Reverse	
β-actin	CATGTACGTTGCTATCCAGGC	CTCCTTAATGTCACGCACGAT	88
GPDH	ACAACTTTGGTATCGTGGAAGG	GCCATCACGCCACAGTTTC	73
RPL13a	TCAAAGCCTTCGCTAGTCTCC	GGCTCTTTTTGCCCGTATGC	104
ASIC1a	CGAAGCAGGCATCAAAGTGC	TTTGGATGATAGGGAGCCACG	642
ASIC2	CACCAAGACTTCACCACAGTGTTT	TGTAGCGGGTCTCACAGTCA	409
ASIC3	TACAAGAACTGTGCCCACCC	GGTCTTCGGAACAGAGCAGA	502
ASIC4	GAGGAGAGAGACAAGCGGCA	GTCCAGCATGATCTCCAGGC	930
α-ENaC	CCAGGCCGCTGCACCT	GCCGATCTTCCAGTCCTTCC	750
γ-ENaC	GAGTGACGTGCCAATCAGGA	TCTCCGAAACCACAGATGGC	305

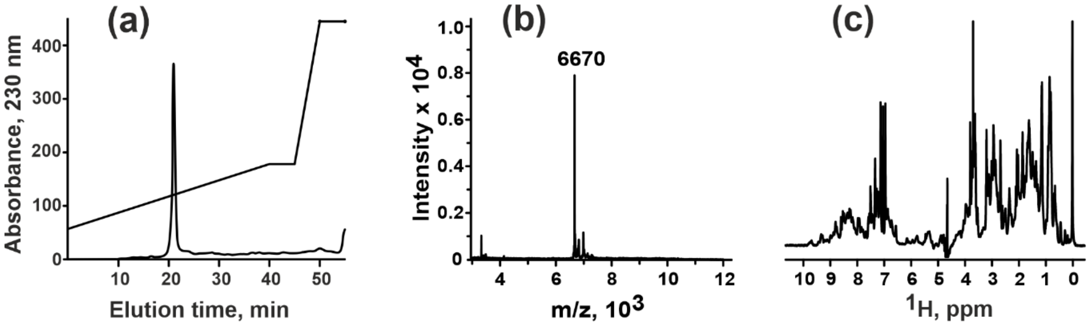

Figure S1. Characterization of the refolded mambalgin-2: (a) HPLC analysis of mambalgin-2 homogeneity and purity; (b) Mass-spectrometry analysis of mambalgin-2; (c) ^{1}H-NMR spectra of mambalgin-2.

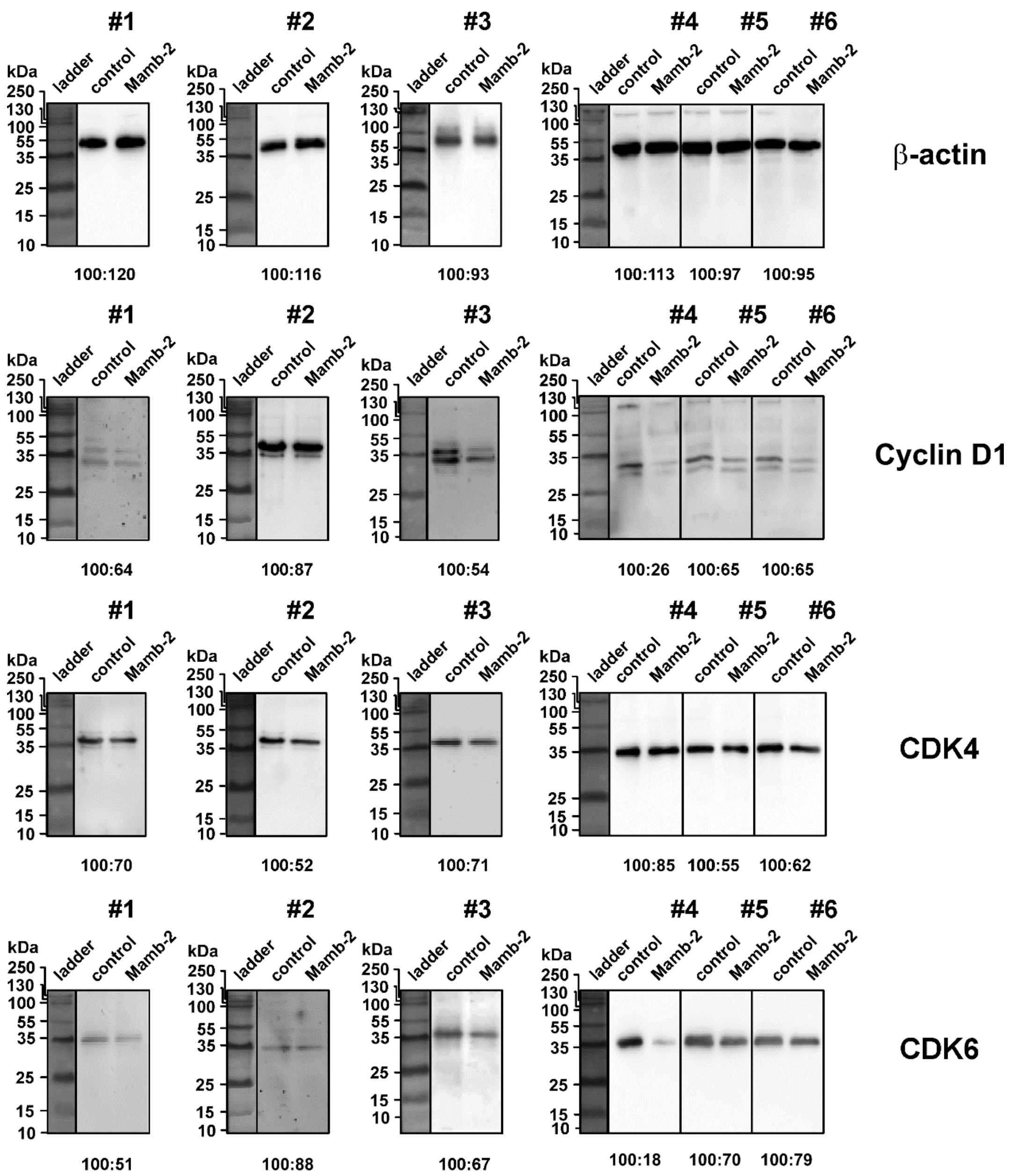

Figure S2. Western blots from 6 independent portions of U251MG cells, showing the mambalgin-2 influence on phosphorylation of Cyclin D1 (pSer90), CDK4 (pThr172), and CDK6 (pTyr24) expression. Cells were incubated with 1 μM mambalgin-2 or 0,1% DMSO for 72 h (see methods) and protein phosphorylation/expression was analyzed by western blot. Cell portions of cells are shown as #1-#6. Portions #1-#3 were analyzed on separate nitrocellulose membranes, portions #4-6 were analyzed on the same membrane. Protein ladder (Thermo Fisher, 26619) from optical channel is shown on the left of each membrane. Optical density ratio of the protein bands corresponded to the untreated cells (control) and mambalgin-2 treated cells (Mamb-2) is presented below the lanes.

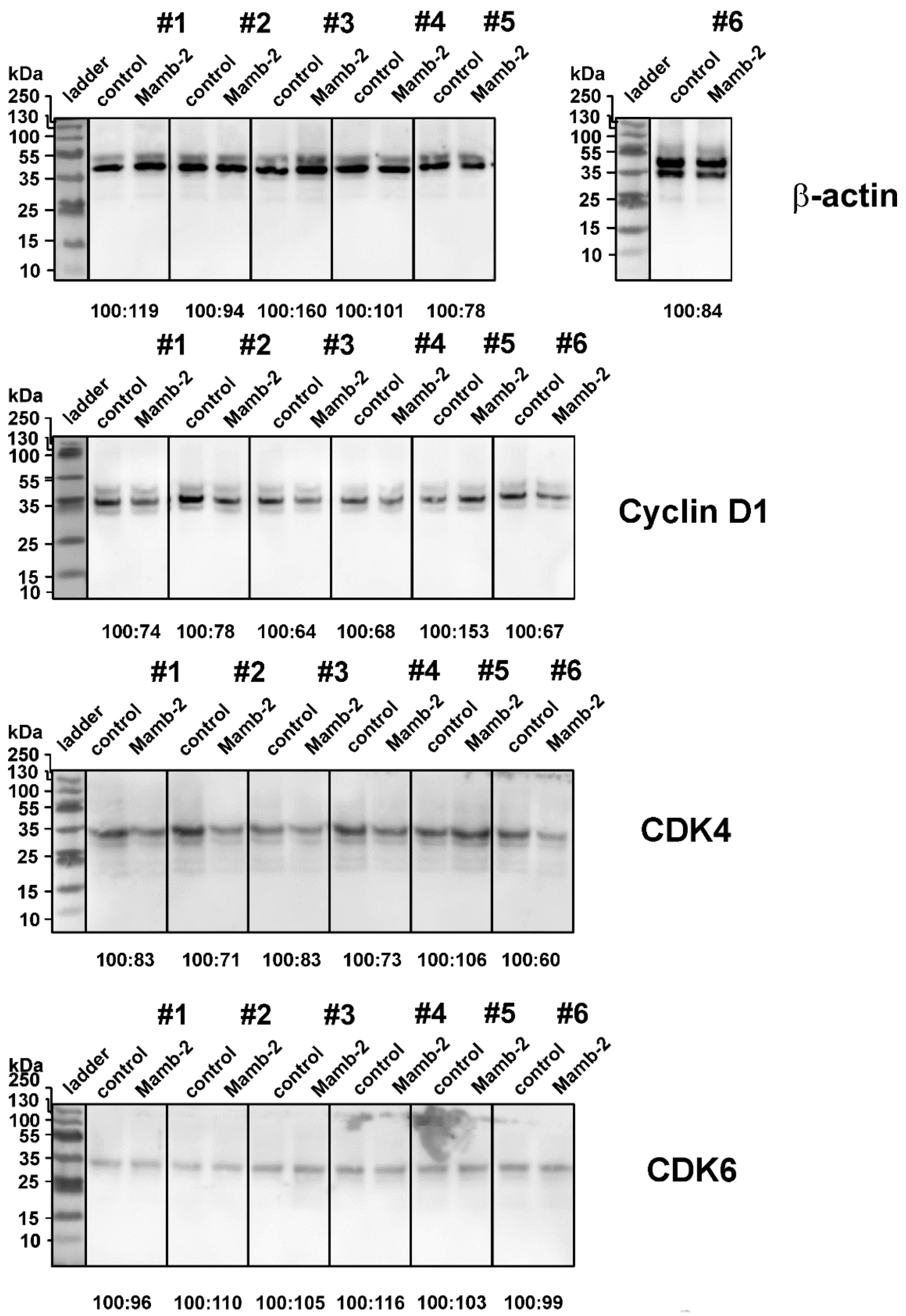

Figure S3. Western blots from 6 independent portions of A172 cells, showing the mambalgin-2 influence on phosphorylation of Cyclin D1 (pSer90), CDK4 (pThr172), and CDK6 (pTyr24) expression. Cells were incubated with 1 μM mambalgin-2 or 0,1% DMSO for 72 h (see methods) and protein phosphorylation/expression was analyzed by western blot. Cell portions of cells are shown as #1-#6 and were analyzed on the same membrane. Protein ladder (Thermo Fisher, 26619) from optical channel is shown on the left of each membrane. Optical density ratio of the protein bands corresponded to the untreated cells (control) and mambalgin-2 treated cells (Mamb-2) is presented below the lanes.

© 2020 by the author. Licensee MDPI, Basel, Switzerland. This article is an open access article distributed under the terms and conditions of the Creative Commons Attribution (CC BY) license (http://creativecommons.org/licenses/by/4.0/).
